# Supplementary figures and images for: Cryo-EM structures of an LRRC8 chimera with native functional properties reveal heptameric assembly
Source: eLife. 2023 Mar 10;12:e82431. doi: 10.7554/eLife.82431 (PMC10049205; doi:10.7554/eLife.82431)

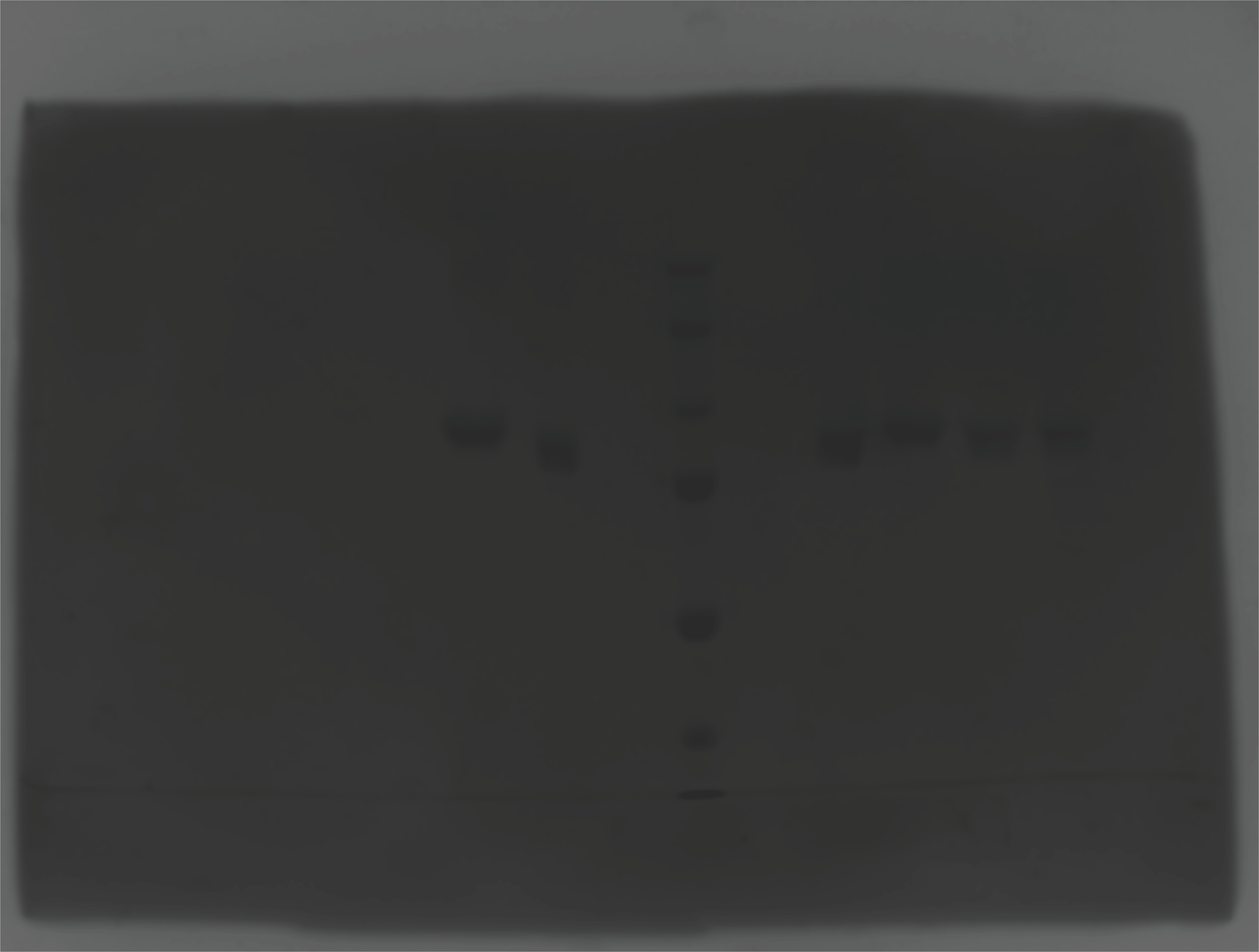

Supplement: Figure 1—figure supplement 1—source data 1. [file elife-82431-fig1-figsupp1-data1.zip › Supplementary_Figure_1_source_data-1/Supp_Fig_1d_source.tif]

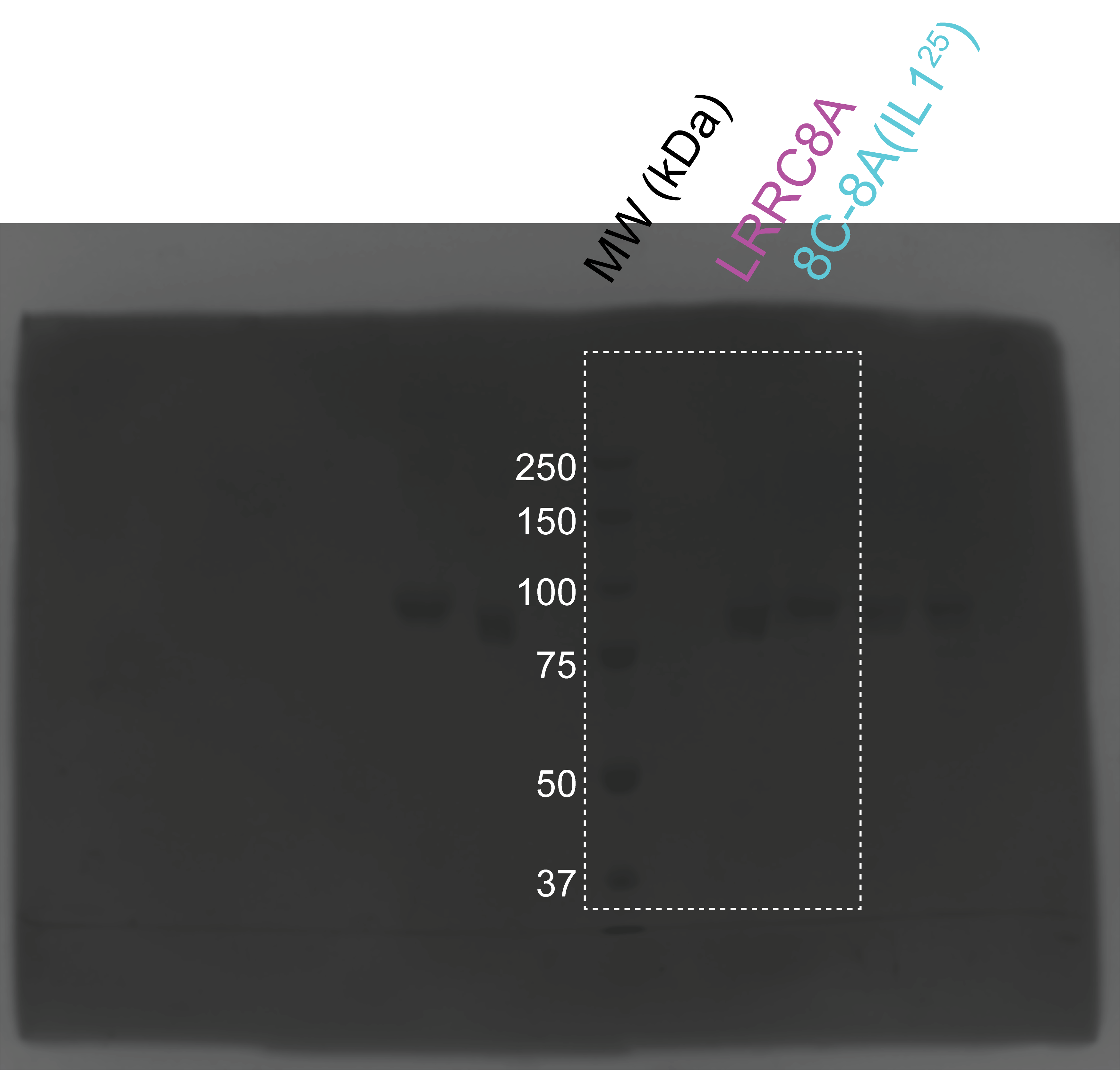

Supplement: Figure 1—figure supplement 1—source data 1. [file elife-82431-fig1-figsupp1-data1.zip › Supplementary_Figure_1_source_data-1/Supp_Fig_1d_source_annotated.tif]

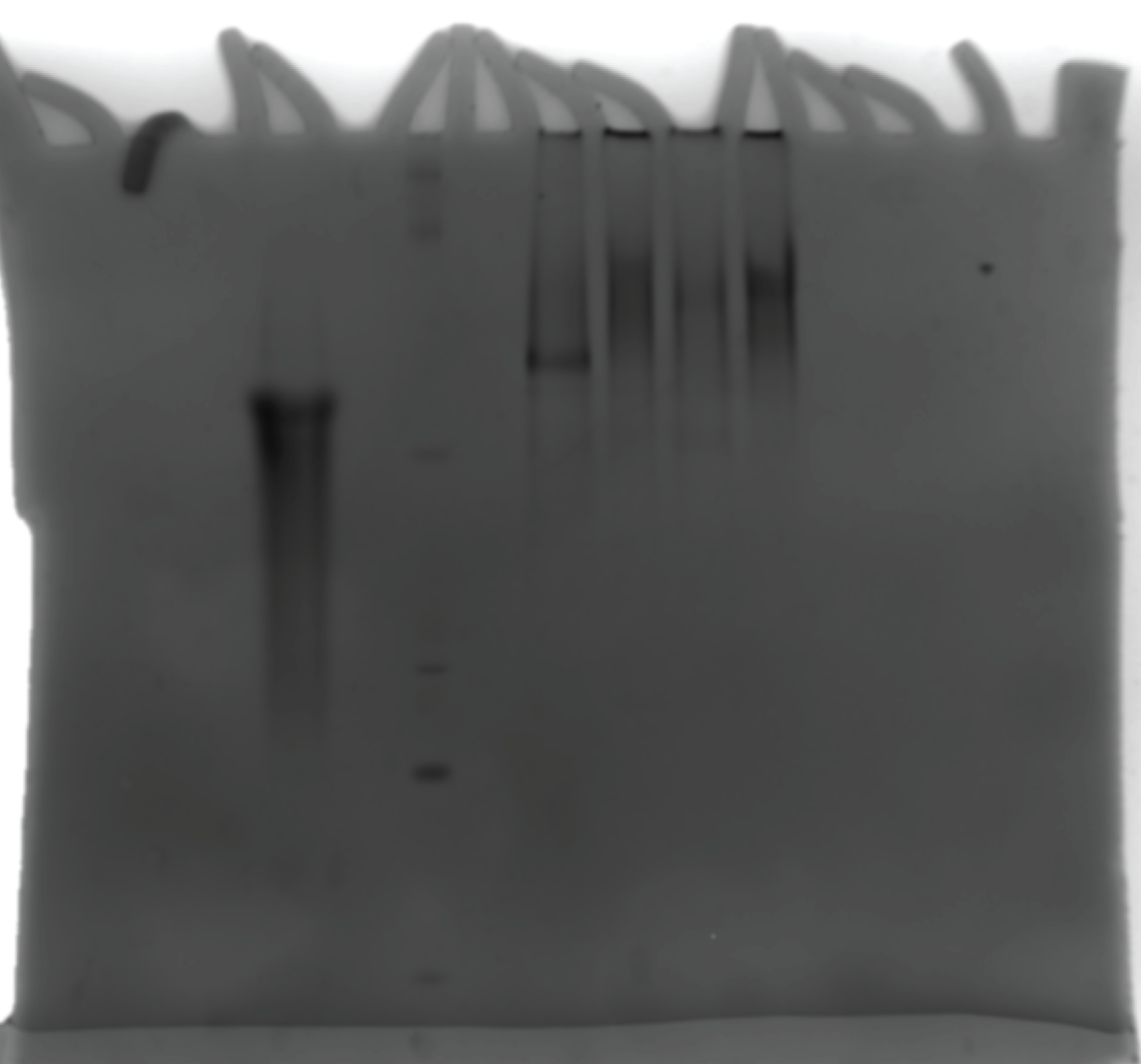

Supplement: Figure 1—figure supplement 1—source data 2. [file elife-82431-fig1-figsupp1-data2.zip › Supplementary_Figure_2_source_data-2/Supp_Fig_1e_source.tif]

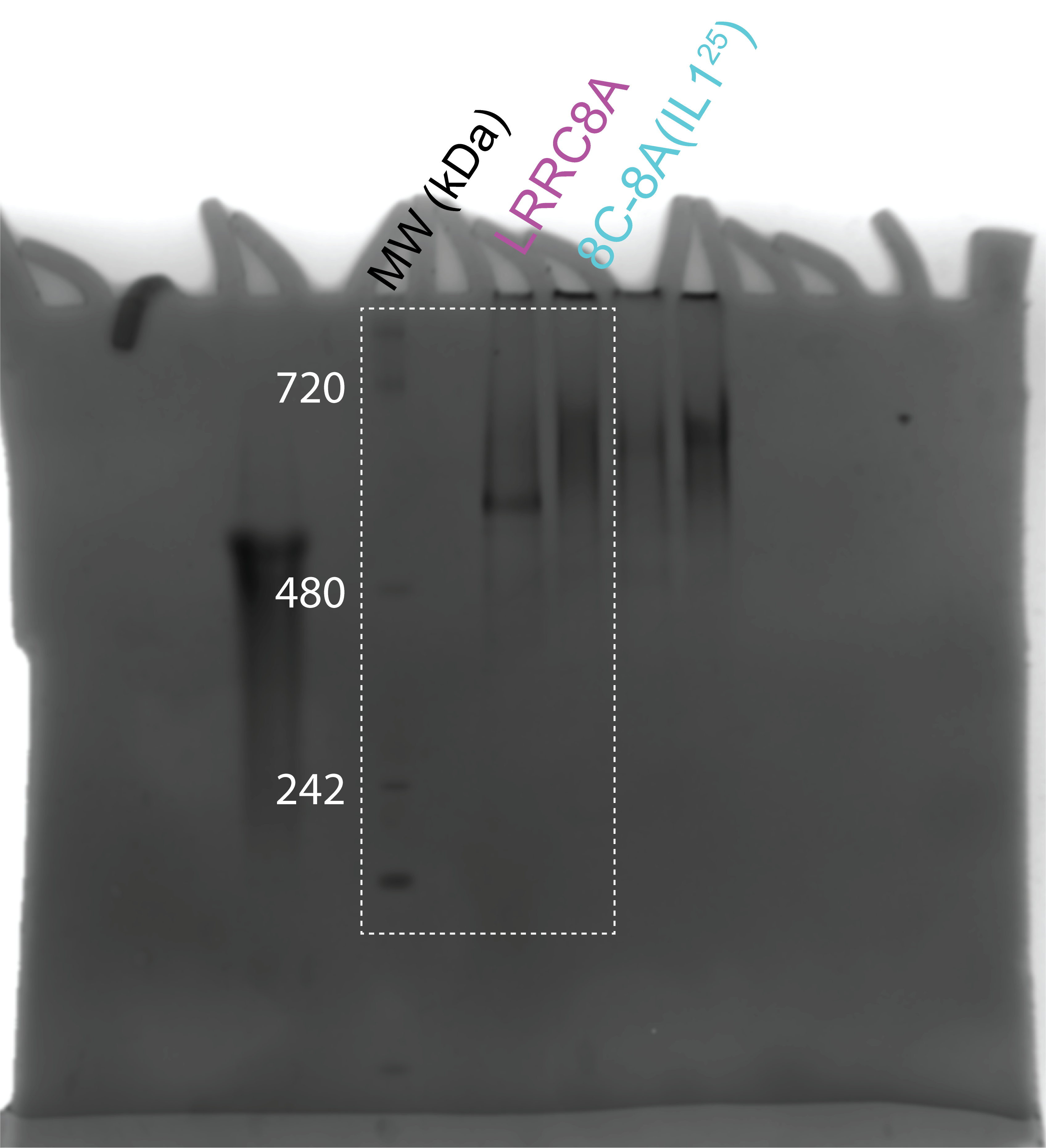

Supplement: Figure 1—figure supplement 1—source data 2. [file elife-82431-fig1-figsupp1-data2.zip › Supplementary_Figure_2_source_data-2/Supp_Fig_1e_source_annotated.tif]
